# What can administrative registers tell us about the widening in life expectancy gap in people with mental disorders?

**DOI:** 10.1192/bjo.2025.10927

**Published:** 2025-12-05

**Authors:** Oleguer Plana-Ripoll, Tomáš Formánek, Natalie C. Momen

**Affiliations:** National Centre for Register-based Research, Department of Public Health, Aarhus University, Aarhus, Denmark; ISGlobal, Barcelona, Spain

**Keywords:** Mortality and morbidity, time trends, register-based epidemiology, psychotic disorders/schizophrenia, bipolar type I or II disorders

## Abstract

In an article published in *BJPsych Open*, a study by Fleetwood and colleagues used Scottish administrative registers to show that not only have people with severe mental illnesses a profoundly reduced average life expectancy compared with the general population, but that the life expectancy gap had been further widening for those with schizophrenia and bipolar disorder over the past 20 years. This study has substantial clinical and public health importance, providing robust evidence to help in evaluation and planning of healthcare services in Scotland. Furthermore, this work raises important questions concerning the study of premature mortality in people with mental disorders per se, as well as the utility of administrative registers to study this phenomenon, which we highlight in this Editorial.

**Figure:**
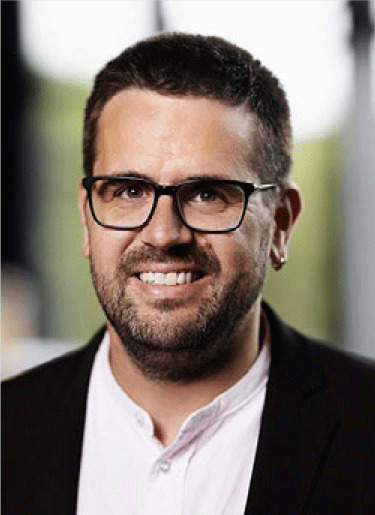

There is robust evidence demonstrating that people with mental disorders die prematurely,^1–3^ driven predominantly by natural causes (i.e. deaths from diseases and medical conditions),^4^ and even when socioeconomic position is taken into account.^5^ In their study published in *BJPsych Open*,^6^ Fleetwood and colleagues used administrative registers to estimate the life expectancy gap for those with severe mental illnesses in Scotland, and explored temporal trends in this gap. Individuals with schizophrenia demonstrated considerable reductions in life expectancy compared with the general population and, worryingly, the life expectancy gap has increased even further over the past decades – from 9.4 excess life-years in 2000–2002 to 11.8 in 2017–2019 for males, and from 8.2 to 11.1 for females. The findings concerning bipolar disorder showed a similar pattern, albeit affected by more uncertainty – increases in excess life-years lost from 6.0 to 7.1 in males and from 5.4 to 6.5 in females. People with depression, while demonstrating considerable reductions in life expectancy, had a broadly consistent life expectancy gap over time (with changes from 7.5 to 7.8 excess life-years lost in males and from 6.6 to 6.5 in females).

The study by Fleetwood and colleagues^6^ adds to the existing knowledge base on premature mortality in people with mental disorders, in two key areas. First, it provides robust and detailed information on the outcomes of people with severe mental illnesses living in Scotland. While there are numerous studies from the UK, the vast majority of these relied on data covering only areas in England – frequently London in particular. Without high-quality, country-specific evidence, the evaluation and planning of services are severely limited – this study helps fill the gap for Scotland. Second, it is one of the few existing studies that has explored changes in mortality over time. Examining temporal trends is crucial to understanding whether the disparities are decreasing, stagnant or even deteriorating. Worryingly, the authors showed that, in less than 20 years, the life expectancy gap has increased appreciably in those with schizophrenia. Evidence from Denmark covering the period 1995–2015 has demonstrated a reduction in life-years lost due to external causes of death (i.e. suicides, accidents and homicides) in people with schizophrenia; however, this was accompanied by an increase in life-years lost due to natural causes of death, meaning no overall improvement.^7^ The Scottish results show an inverse pattern, with decreases in natural causes and small increases in external causes of death. While the trend related to deaths from natural causes suggests positive developments, the findings for external causes of death are concerning – additional research would be warranted to dissect the factors responsible for these findings, and to investigate the reasons for differing trends between countries.

In addition to providing essential evidence of substantial clinical and public health importance, the work of Fleetwood et al^6^ raises a number of crucial questions concerning the study of premature mortality, which we would like to highlight. First, while most studies use measures of relative differences such as mortality rate ratios or standardised mortality ratios to explore excess mortality, this study focuses on life expectancy. The use of a measure of absolute difference – expressed in commonly understood units (i.e. years) – facilitates interpretation of the findings from a public health perspective. However, life expectancy is a measure based on mortality rates over all different ages, and thus it does not allow identification of particular periods over the life-course contributing to the life expectancy gap or changes in it. Integration of absolute and relative measures – including mortality rate ratios at specific ages – has the potential to provide more penetrating, in-depth understanding of the studied phenomenon.^4^ Second, the measure of life expectancy used by the authors – based on the life-years-lost method^8,9^ – takes into account the observed age-at-diagnosis distribution, contrasting with many previous studies that used a fixed age at diagnosis (typically 15 years). Thus, considering the underlying age-at-diagnosis structure led to more plausible estimates than those previously reported. Nevertheless, in regard to the delay between disease onset and diagnosis in routine care, it is important to note that the life-years-lost method would probably contribute to underestimation of the true burden. Third, during the observed period, the authors included cases of mental disorders that were both prevalent (diagnosed since 1981 and before the start of the follow-up in 2000) and incident (diagnosed during the follow-up). Excess mortality is, on average, higher in the immediate years following the diagnosis of a mental disorder than later on, mostly driven by the risk of dying from suicide.^10,11^ Thus, the methodological choice of including prevalent cases – who had to survive an initial period following the mental disorder diagnosis – has probably had important consequences for the study results. Restricting the analyses to incident cases would arguably have resulted not only in the life expectancy gap being even more pronounced, but the widening of such a gap would have also been potentially greater: the 2000–2002 period included prevalent cases diagnosed up to 19 years earlier, while the 2017–2019 period included prevalent cases diagnosed up to 36 years earlier, for which excess mortality might have been even lower after having survived such a long period. Future studies should consider performing sensitivity analyses restricted to incident cases of mental disorders and/or using a similar length of look-back periods when assessing changes over time. Finally, the study used data covering in-patient services, and it can be assumed that it consistently covered the most severe cases of the studied mental disorders. However, deinstitutionalisation and related efforts would arguably pressure in-patient services to focus on increasingly more severe cases.^12^ Differences in case severity over time could then have impacted the estimates. This phenomenon would be, nevertheless, even more pronounced when using data covering different segments of the healthcare system (primary and out- and in-patient services), considering that the potential time trends in diagnostic practices might be captured as well.

In summary, the mortality gap experienced by people with mental disorders remains a major challenge for medicine and public health.^13^ The recent Gone Too Soon framework^14^ identified 18 actionable solutions across 3 organising principles to reduce premature mortality from both natural and external causes. These principles, which we would like to reiterate, include (a) providing mental and physical health care in an integrated manner, (b) focusing on prevention while simultaneously ensuring effective and accessible treatment and (c) facilitating intervention synergies accounting for various factors across structural, community, relationships and individual levels.^14^ Analyses of data from administrative registers have already played a key role in highlighting the disparity in life expectancy among those with mental disorders. However, the mortality gap still exists and, as the study by Fleetwood and colleagues has demonstrated,^6^ it may be even increasing for some populations. We argue that further epidemiological research is needed to help in identifying sociodemographic and clinical subgroups at the highest risk of mortality. Administrative registers, often covering large populations, provide readily available data that allow interrogations of this kind. Additionally, the continuous collection of data can help to reveal patterns over increasingly long time periods, including following the introduction or modification of policies concerning people with mental disorders. We also argue that this epidemiological work should, preferably, be accompanied by further methodological investigations assessing how the coverage and quality of data in administrative registers impact mortality estimates. In particular, the consistency of results based on different segments of the healthcare system (primary and out- and in-patient services), and the validity of recorded diagnoses, warrant more attention.
